# Effects of *p*-Synephrine during Exercise: A Brief Narrative Review

**DOI:** 10.3390/nu13010233

**Published:** 2021-01-15

**Authors:** Carlos Ruiz-Moreno, Juan Del Coso, Verónica Giráldez-Costas, Jaime González-García, Jorge Gutiérrez-Hellín

**Affiliations:** 1 Exercise Physiology Laboratory, Camilo José Cela University, 28693 Villanueva de la Cañada, Spain; cruizm@ucjc.edu (C.R.-M.); vgiraldez@ucjc.edu (V.G.-C.); jgonzalez@ucjc.edu (J.G.-G.); 2 Centre for Sport Studies, Rey Juan Carlos University, 28943 Fuenlabrada, Spain; 3 Faculty of Health Sciences, Francisco de Vitoria University, 28223 Pozuelo, Spain; jorge.gutierrez@ufv.es

**Keywords:** alkaloids, body composition, carbohydrate sparing, weight loss, exercise

## Abstract

The *p*-synephrine is the principal phytochemical found in bitter orange (*Citrus aurantium*). This substance is widely included in dietary supplements for weight loss/body fat reduction due to its potential benefits of increasing fat oxidation. For years, *p*-synephrine-containing dietary supplements have been marketed without proper knowledge of their true effectiveness to enhance fat utilization, especially when combined with exercise. However, the effects of *p*-synephrine on fat oxidation during exercise have been investigated in the last few years. The aim of the current discussion is to summarize the evidence on the effects of *p*-synephrine intake on fat oxidation and performance during exercise. Previous investigations have demonstrated that the acute intake of *p*-synephrine does not modify running sprint performance, jumping capacity, or aerobic capacity. However, the acute intake of *p*-synephrine, in a dose of 2–3 mg/kg of body mass, has been effective to enhance the rate of fat oxidation during incremental and continuous exercise. This effect has been observed in a range of exercise workloads between 30% and 80% of peak oxygen uptake (VO_2peak_). The *p*-synephrine has the ability to increase the maximal rate of fat oxidation during exercise of increasing intensity without affecting the workload at which maximal fat oxidation is obtained (Fatmax). The effect of *p*-synephrine on fat oxidation is normally accompanied by a concomitant reduction of carbohydrate utilization during exercise, without modifying the energy expended during exercise. The shifting in substrate oxidation is obtained without any effect on heart rate during exercise and the prevalence of adverse effects is negligible. Thus, the acute use of *p*-synephrine, or *p*-synephrine-containing products, might offer some benefits for those individuals seeking higher fat utilization during exercise at low to moderate intensities. However, more research is still necessary to determine if the effect of *p*-synephrine on fat oxidation during exercise is maintained with chronic ingestion, in order to ascertain the utility of this substance in conjunction with exercise programs to produce an effective body fat/weight loss reduction.

## 1. Introduction

The *p*-synephrine (4-[1-hydroxy-2-(methylamino)ethyl]phenol) is a phenylethylamine derivative present in the fruits of several trees from *Rutaceae* family such as Bitter Orange (*Citrus aurantium*), Seville Orange, Sour Orange, Green Orange, Zhi Shi, and Kijitsu. Additionally, this phytochemical can be found in many other citrus species such as Nova tangerines and Marr’s sweet oranges [1]. In its natural state, *p*-synephrine is the main phytochemical in most of these fruits, and it is present in the range of 0.10–0.35% in fruits of *Citrus aurantium* and in the range of 3.00–3.08% in dry extracts of this fruit [2]. However, the amount of *p*-synephrine can be artificially increased by concentrating the extract of natural products, and *p*-synephrine may be in a concentration of up to 19% in some commercially available dietary supplements [3]. This allows the intake of moderate amounts of *p*-synephrine in one capsule or scoop of dietary supplements without the need of ingesting a large amount of fruit. Interestingly, *p*-synephrine became popular as an active ingredient for thermogenics and weight-loss supplements due to the ban of *Ephedra* species by the U.S. Food and Drug Administration in 2004 [4]. Several dietary supplements companies substituted *Ephedra* with *Citrus aurantium* or *p*-synephrine because they purportedly have the capacity to increase the metabolic rate at rest and enhance lipolysis [2,5]. It is believed that, in the long term, the chronic ingestion of *p*-synephrine may reduce fat mass through increased thermogenesis and fat oxidation, although there is no clinical or research evidence to support this notion by using supplements containing only *p*-synephrine. Despite the lack of evidence, *p*-synephrine is widely present in slimming, weight loss, thermogenics, and supplements for meal replacement and its presence in the market of weight-loss products is comparable to other stimulants with well-contrasted efficacy such as caffeine [6]. In the last few years, some investigations have aimed to determine the efficacy of *p*-synephrine to enhance fat utilization during exercise. This knowledge may be essential for those individuals seeking body fat reduction as the participation in regular exercise is one of the best practices to produce effective body composition changes. Understanding the additive action of *p*-synephrine intake and exercise may help to integrate *p*-synephrine as weight-loss supplement in exercise programs for weight and obesity management [7]. For these reasons, the aim of the current discussion is to summarize the existing evidence on the effects of *p*-synephrine intake on substrate oxidation, energy expenditure, and cardiovascular variables during exercise. With this discussion, we aim to shed light on the utility of this substance to enhance fat mass loss when used in combination with exercise.

## 2. Chemical Structure of *p*-Synephrine

Synephrine is an unspecific adrenergic agonist, which can exist in three different positional isomers (ortho *o*-, para *p*-, and meta *m*-) [8]. The *m*-synephrine and *o*-synephrine do not occur naturally in *Citrus aurantium* and they are not habitually present in plants’ extracts [9]. The *m*-isoform, also named phenylephrine, is considered the most potent synephrine adrenergic agonist at α-1 adrenoceptors, when compared to the other positional isomers [10]. The *o*-synephrine isoform has no pharmacological effect in humans and it has not been found in dietary supplements [11]. The subtle structural and stereochemical differences of *p*-synephrine and *m*-synephrine result in different adrenergic receptor binding characteristics that lead to mechanistic differences. Specifically, the absence of adrenergic receptor binding of *p*-synephrine provides the explanation for its lack of production of harmful cardiovascular effects as compared to *m*-synephrine. Hence, while *m*-synephrine can be considered as an alkaloid with a potent cardiovascular effect [12], *p*-synephrine or Citrus aurantium intake are normally not associated to harmful cardiovascular events [13].

Additionally, each positional isomer can also be found in two enantiomeric forms with different pharmacological and physiological activities. For example, *p*-synephrine exists in nature in the l- or [R-(−)]-enantiomeric form, whereas synthetic *p*-synephrine like *synephrine hcl* is a racemic mixture of the l- and d-enantiomeric forms. Based on receptor binding, the synthetic form is believed to exert approximately half the pharmacological activity of the naturally occurring *p*-synephrine [14]. This difference occurs because the d- or [S-(+)]-form provides little or no binding to adrenergic receptors in contrast to the l-form [14]. This is important because a recent report suggests that most dietary supplements containing *p*-synephrine or *Citrus aurantium* do not contain the plant extract [15]. Therefore, the efficacy of commercially available supplements may be reduced when compared to natural products or plants’ extracts for the same amount of *p*-synephrine.

## 3. Binding Capacity and Mechanisms of Action

The purported effects of *p*-synephrine to enhance thermogenesis and fat oxidation at rest and during exercise were based on its structural similarity with endogenous amine neurotransmitters such as epinephrine and norepinephrine [11]. Due to this structure, *p*-synephrine has the ability to bind β-3 adrenoceptors [16]. As a result, *p*-synephrine increases lipolysis at rest [17,18], although the evidence of enhanced lipolysis during exercise is lacking [18]. The β-3 adrenoceptors are expressed in human white as well as brown adipose tissue and in skeletal muscle, and they play a role in the regulation of energy balance and glucose and fat homeostasis [19]. Therefore, *p*-synephrine may increase resting metabolic rate and energy expenditure, although most of this evidence in humans has been found when using dietary supplements containing p-synephrine in addition to other co-ingredients [20]. Additionally, *p*-synephrine elicits an efficient stimulation of beige adipocyte differentiation through β-3 stimulation with the support of several beige adipocyte differentiation-inducing factors [21]. Hence, β-3 activation induced by *p*-synephrine may be key to enhance fat utilization, especially in the skeletal muscle where a large amount of fat can be used to provide energy for contraction. On the other hand, *p*-synephrine has a low binding affinity for α- and β-1 and β-2 adrenoreceptors. In comparison to norepinephrine, the binding of *p*-synephrine has been reported to be about 40,000-fold less to β-1 and β-2 adrenergic receptors and approximately 1000-fold less to α-1 and α-2 adrenergic receptors [16]. The lack of α-1, α-2, β-1, and β-2 adrenergic receptor binding supports the lack of effects of *p*-synephrine on heart rate and blood pressure [20], which translates into a low rating of cardiovascular and hemodynamic side effects derived from acute and chronic *p*-synephrine ingestion.

Furthermore, *p*-synephrine can stimulate specific receptors in the brain. The *p*-synephrine is a highly potent and selective agonist of Neuromedin U2 receptors (NMU2R). Specifically, NMU2R is prominent in the hypothalamic regions and is known to be associated with regulation of several important physiological functions, including food intake, energy balance, stress response, and nociception [22]. The activation of β-3 adrenergic receptor and NMU2R may contribute to the lipolytic activity of *p*-synephrine and it is believed that *p*-synephrine may aid in the regulation of food intake and energy homeostasis by active NMU2R on the central nervous system. In experiments carried out with animals, in which the isolated rat liver was perfused in vivo after oral administration of *p*-synephrine, it has been found that *p*-synephrine has a potent capacity to inhibit the pyruvate dehydrogenase enzyme [23]. The decrease in the activity of the pyruvate dehydrogenase enzyme indicates that *p*-synephrine can inhibit the transformation of carbohydrates into lipids, which may also add to body fat homeostasis. Lastly, *p*-synephrine suppresses the 3T3-L1 cell adipogenesis by reducing the expression level of CCAAT-enhancer-binding protein α (C/EBPα) and peroxisome proliferator-activated receptor γ (PPARγ), which subsequently leads to a reduction in the fatty acid-binding protein 4 (aP2) expression. Specifically, different treatments with *p*-synephrine activate the protein kinase B (PKB/Akt) pathway and sequentially inhibits glycogen synthase kinase 3β (GSK3β) activity [24]. The Akt signaling pathway plays a pivotal role in modulating adipogenesis while GSK3β is a crucial protein involved in cell signaling [25]. The Akt phosphorylation (activation) and the GSK3β phosphorylation (inactivation) induced by *p*-synephrine administration reduced the expression of PPARγ, a nuclear receptor protein with an essential role in the regulation of adipocyte differentiation. As a consequence of these effects, *p*-synephrine inhibited adipogenesis and the differentiation of adipocyte in 3T3-L1 preadipocytes. Collectively, this information suggests that *p*-synephrine may exhibit anti-adipogenic effects via different signaling pathways, the suppression of adipogenesis-related proteins, and improved regulation of energy balance and food intake.

## 4. Ergogenic Effect of *p*-Synephrine during Exercise

The structural similarity of *p*-synephrine with endogenous amine neurotransmitters was the ground to hypothesize that this substance may increase exercise performance through enhanced stimulation of the sympathetic nervous system [16]. However, as we saw above, the characteristics of this alkaloid are different to other similar endogenous amine due to low binding to adrenergic receptors. Despite this, *p*-synephrine was added to the Monitoring Program of the World Anti-Doping Agency (WADA) in 2009 [26] because it has been considered as a substance with the potential of increasing exercise and sports performance. The Monitoring Program was designed to assess the trends in the use of substances that have the potential of being included in the WADA’s prohibited list. However, substances in the Monitoring Program are not officially banned in sport because the evidence to ascertain their efficacy to increase performance while affecting an athlete’s health is not definitive. This means that *p*-synephrine has never been a banned substance, but it is still included in the 2020 WADA Monitoring Program [26]. To date, the evidence is pointing toward the lack of ergogenic activity of *p*-synephrine during exercise and sport, although studies to demonstrate an increased exercise capacity after acute *p*-synephrine intake are scarce and contradictory. For example, the acute intake of *p*-synephrine (3 mg/kg of body mass) did not change running performance during 60-m and 100-m sprint races and it did not affect single and repeated jumping capacity of sprinters when compared to a placebo [27]. In addition, the ingestion of the same dose of *p*-synephrine did not increase peak wattage nor the maximum oxygen uptake (VO_2max_) obtained during a ramp exercise test in elite cyclists [28]. The only evidence demonstrating an increase in performance with *p*-synephrine is a study reporting a higher capacity to perform squat repetitions during six sets of exercise with a load equivalent to 80% of participants’ one-repetition maximum [29]. In this latter experiment, *p*-synephrine was ingested in a dose of ~1 mg/kg of body mass for three days before the resistance exercise protocol. Further investigations are required to clearly determine the ergogenic capacity of *p*-synephrine, which will help anti-doping authorities decide if the substance should be included in the list of banned substances or if it its should be stopped. To the authors’ opinion, the lack of ergogenic effect of this substance during exercise, together with the low prevalence of adverse effects, discussed below, does not support the inclusion of *p*-synephrine in the WADA Monitoring Program.

## 5. Effect of *p*-Synephrine to Shifting Substrate Oxidation during Exercise

**Figure 1:**
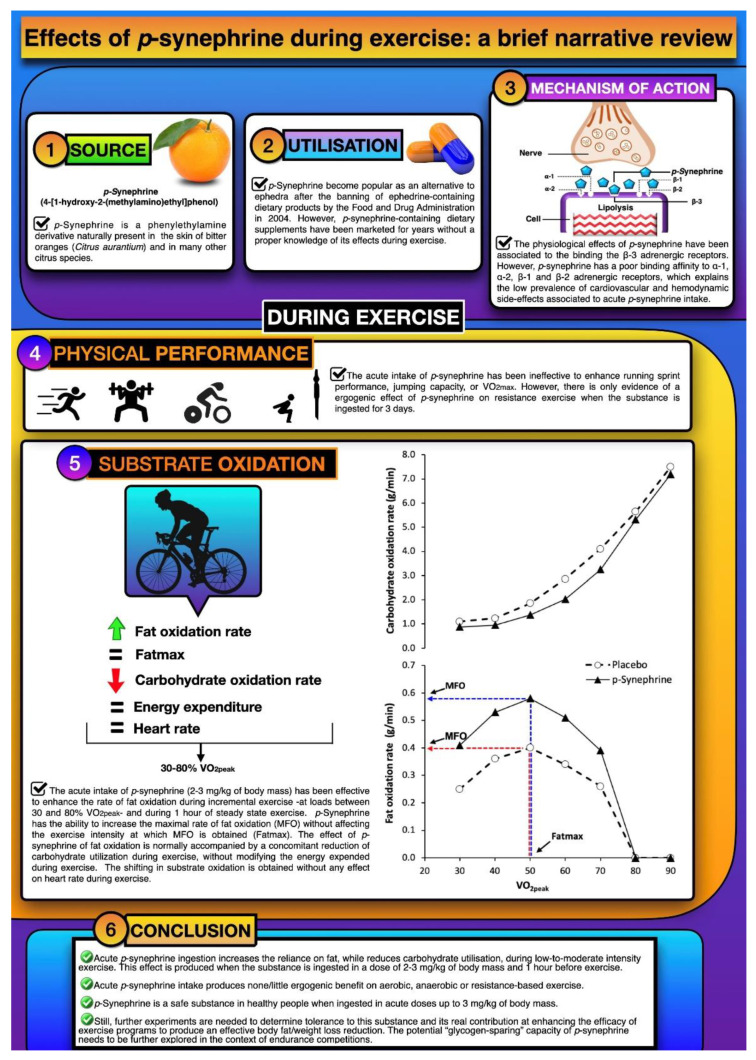
Infographic with a visual explanation of *p*-synephrine. The *p*-synephrine is a phenylethylamine derivative naturally present in the skin of bitter oranges (*Citrus aurantium*) and in many other citrus species. The *p*-synephrine became popular as an alternative to ephedra after the banning of ephedrine-containing dietary products by the Food and Drug Administration in 2004. The physiological effects of *p*-synephrine have been associated to the binding with the β-3 adrenergic receptors. During exercise the acute intake of *p*-synephrine has been ineffective to enhance performance. Acute intake of *p*-synephrine (2–3 mg/kg of body mass) has been effective to enhance the rate of fat oxidation during incremental exercise at loads and during 1 h of steady-state exercise. The *p*-synephrine has the ability to increase the maximal rate of fat oxidation without affecting the exercise intensity at which maximal fat oxidation (MFO)is obtained (i.e., Fatmax).

Despite the wide popularity of *p*-synephrine in the weight-loss supplement market, the efficacy of this substance to enhance fat oxidation during exercise has only been demonstrated in the last few years (Figure 1). A first report in 2016 demonstrated that 3 mg of *p*-synephrine/kg of body mass significantly enhanced fat oxidation during a cycling test of increasing intensity in healthy individuals [5]. In this investigation, the acute intake of *p*-synephrine increased the maximal rate of fat oxidation from 0.29 to 0.40 g/min while it did not affect the exercise intensity at which maximal fat oxidation was achieved (~56% VO_2max_). Similar findings have been obtained in elite cyclists with the same dose, but, in this case, the *p*-synephrine induced a change in the rate of maximal fat oxidation from 0.91 to 1.01 g/min, again without affecting Fatmax [28]. It is well established that aerobically trained individuals have higher rates of fat oxidation during exercise than untrained individuals [30] due to the different adaptations that aerobic training induces in the cardiorespiratory system and within the skeletal muscle. The comparison of these two studies [5,28] suggests that acute *p*-synephrine intake has the capacity of increasing the rate of fat utilization during exercise irrespective of the fitness level of the individual. Hence, it seems that the adaptations induced by aerobic training do not prevent the benefits of acute *p*-synephrine intake on fat oxidation during exercise. Additionally, it has been found that there is needed ingesting of at least 2 mg/kg of body mass of *p*-synephrine to significatively increase the rate of fat oxidation during exercise of increasing intensity, while it sems that the effect of this substance on fat oxidation plateaus at 3 mg/kg of body mass [31]. In all these investigations [5,28,31], the acute intake of *p*-synephrine moves upwards the fat oxidation–exercise intensity curve and the effect of *p*-synephrine was present at several exercise intensities between 30% and 80% of VO_2max_. As a result, it seems safe to indicate that *p*-synephrine is effective to enhance fat utilization during exercise of low to moderate intensity. In fact, it has been recently found that 3 mg/kg of body mass of *p*-synephrine increased the amount of fat oxidized during 1 h of exercise at Fatmax, equivalent to ~57% VO_2max_ from 33.6 to 37.3 ± 9.8 g [32]. Interestingly, the co-ingestion of *p*-synephrine and caffeine does not produce a synergistic effect on fat oxidation during exercise over the benefit of these substances when they are ingested isolatedly [33]. In addition, it has been found that the magnitude of the effect of *p*-synephrine on fat oxidation during exercise of increasing intensity [33] and during steady-state exercise is similar to caffeine when both substances are ingested in a dose of 3 mg/kg of body mass [32,34]. Collectively, this evidence supports the benefit of acute *p*-synephrine intake to increase the rate of fat oxidation during low- to moderate-intensity exercise. Although the magnitude of the effect of this substance is small (increase of ~0.1 g of oxidized fat per min of exercise), the use of *p*-synephrine-containing supplements may be an option for those individuals seeking to maximize fat oxidation during endurance exercise. Additionally, *p*-synephrine may also increase the rate of fat oxidation after exercise, although this effect requires further research [18].

In all the abovementioned investigations, the increases in fat oxidation rate with *p*-synephrine were accompanied by a concomitant reduction in carbohydrate oxidation rates [28,30,31,32]. This is because *p*-synephrine does not modify energy expenditure rates at any exercise intensity, which contradicts its potential thermogenic effect, at least during exercise. Hence, it seems that *p*-synephrine produces a shifting in substrate oxidation during exercise without affecting the energy expended with exercise [32]. The reduction of carbohydrate oxidation is evident during exercise of increasing intensity [28] and during steady-state exercise [32], and it may help individuals to spare muscle and liver glycogen during exercise. Glycogen sparing through feeding or training with low carbohydrate availability has been found effective to increase some forms of aerobic exercise performance [35]. However, suggesting that *p*-synephrine may enhance aerobic exercise performance via glycogen sparing is speculative. In any case, this potential ergogenic effect of *p*-synephrine would be associated to exercise or sports performed of long duration and performed at low to moderate intensity as this is the range of exercise intensities where *p*-synephrine exerts the shifting in the substrates oxidized during exercise.

It is important to highlight that the capacity of *p*-synephrine to shifting substrate oxidation during exercise is produced without affecting heart rate [28,30,31,32], although a reduction in perceived exertion has been reported [36]. This is important from a practical view because it concedes to *p*-synephrine the capacity of increasing fat utilization during exercise with minimal changes in the internal load of participants. Therefore, *p*-synephrine can be used during exercise programs aimed to reduce fat mass without the need of modifying exercise intensity or the use of heart rate as a determinant of exercise intensity. Last, the mechanism underlying the capacity of p-synephrine to shifting fuel utilization during exercise is not clear, although the increased lipid availability by greater *p*-synephrine-induced lipolysis in adipose tissue [17,18] is believed to be the main mechanism driving a higher reliance on fat during exercise. However, further experiments assessing *p*-synephrine-induced changes in blood and muscle tissue during exercise are needed to unveil the exact mechanism(s) associated to the benefits of *p*-synephrine.

## 6. Adverse Side Effects with Acute and Chronic Ingestion of *p*-Synephrine

The acute intake of *p*-synephrine in healthy humans has not been associated with side effects, and the safety of this substance is certified by studies with animals with doses of up to 1000 mg/kg/day [37]. In a recent review that summarizes all published case reports about adverse events in humans associated with dietary supplements containing *p*-synephrine, the authors found that, in all cases, the presence of *p*-synephrine was not linked to the cause of the adverse effect [20]. To this regard, by using a double-blind and placebo-controlled study, healthy individuals given *Citrus aurantium* extract with 49 mg *p*-synephrine twice a day for 60 days had no adverse effects with respect to heart rate, blood pressure, blood chemistries, or blood cell counts [38]. Additionally, the intake of 49 mg of *p*-synephrine for 15 days produced no significant changes in heart rate, electrocardiograms, or systolic or diastolic blood pressures in healthy individuals [39]. These well-controlled experiments suggest the safety of chronic *p*-synephrine/*Citrus aurantium* intake, at least up to two months, when ingested in a dose of ~1 mg/kg. Other randomized experimental research hA evaluated the incidence of adverse effects in healthy and active individuals following acute intake of ~1–3 mg/kg of *p*-synephrine and there was no increase in ratings of headaches, gastrointestinal discomforts, muscle pain, or insomnia when compared to a placebo [27,33] nor an impact on blood parameters [40]. Finally, the addition of *p*-synephrine to caffeine does not produce an additional effect on blood pressure or heart rate beyond the effect of caffeine alone [41]. All this information suggests that *p*-synephrine/*Citrus aurantium* are safe substances for acute and chronic ingestion in doses up to 1–3 mg/kg of body mass. Nevertheless, it is important to inform potential users of thermogenics and weight-loss products that other harmful stimulants may be included in commercially available dietary supplements, even when there is no information of the inclusion of these potentially dangerous substances on the label. The authenticity/quality assurance of dietary supplements containing *p*-synephrine [15], especially in those with a multi-ingredient formula, is key to avoiding potential side effects. The use of dietary supplements with pure *p*-synephrine is recommended to obtain its benefits on fat oxidation while reducing the likelihood of harmful effects due to the inclusion of other stimulants.

## 7. Conclusions

In summary, acute *p*-synephrine ingestion increases the reliance on fat, while reducing carbohydrate use as fuel during low- to moderate-intensity exercise. The enhancement in fat utilization with *p*-synephrine is normally obtained without any changes in Fatmax, likely due to the lack of this substance to increase exercise performance. The *p*-synephrine also reduces the rate of carbohydrate oxidation during exercise of low to moderate intensity while energy expenditure and heart rate are generally unchanged. The effect of *p*-synephrine to shift substrate oxidation has been found during ramp exercise tests and during steady-state exercise.

From a practical perspective, the acute use of *p*-synephrine might offer some benefits for those individuals seeking higher fat utilization during exercise. The use of this phytochemical, at doses of up to 3 mg/kg, offers some benefits over other substances such as caffeine due to the lower rate of side effects associated with the former. Although the magnitude of the effect p-synephrine on fat oxidation during exercise is small (increase of fat oxidation ~0.1 g/min), the use of *p*-synephrine-containing supplements may be an option to maximize fat oxidation. However, more research is necessary to determine if the effect of *p*-synephrine on fat oxidation during exercise is maintained with chronic ingestion, in order to ascertain the utility of this substance in conjunction with exercise programs to produce an effective body fat/weight loss reduction. Lastly, more translational research is also necessary to ascertain whether the lower carbohydrate use with *p*-synephrine produces significant glycogen sparing that may be effective to enhance performance in endurance sports competition.

## Data Availability

No new data were created or analyzed in this study. Data sharing is not applicable to this article.

## References

[1] Pellati F., Benvenuti S. (2007). Fast high-performance liquid chromatography analysis of phenethylamine alkaloids in Citrus natural products on a pentafluorophenylpropyl stationary phase. J. Chromatogr. A.

[2] Pellati F., Benvenuti S., Melegari M. (2004). High-performance liquid chromatography methods for the analysis of adrenergic amines and flavanones in *Citrus aurantium* L. var. amara. Phytochem. Anal..

[3] Avula B., Upparapalli S.K., Navarrete A., Khan I.A. (2005). Simultaneous quantification of adrenergic amines and flavonoids in *C. aurantium*, various Citrus species, and dietary supplements by liquid chromatography. J. AOAC Int..

[4] National Institutes of Health Office of Dietary Supplements Ephedra. https://ods.od.nih.gov/HealthInformation/Ephedra.aspx.

[5] Gutiérrez-Hellín J., Del Coso J. (2016). Acute p-synephrine ingestion increases fat oxidation rate during exercise. Br. J. Clin. Pharmacol..

[6] Müller L.S., Moreira A.P.L., Muratt D.T., Viana C., De Carvalho L.M.H. (2019). An ultra-high performance liquid chromatography-electrospray tandem mass spectrometric method for screening and simultaneous determination of anorexic, anxiolytic, antidepressant, diuretic, laxative and stimulant drugs in dietary supplements marketed for weight loss. J. Chromatogr. Sci..

[7] Keating S.E., Johnson N.A., Mielke G.I., Coombes J.S. (2017). A systematic review and meta-analysis of interval training versus moderate-intensity continuous training on body adiposity. Obes. Rev..

[8] Haaz S., Fontaine K.R., Cutter G., Limdi N., Perumean-Chaney S., Allison D.B. (2006). Citrus aurantium and synephrine alkaloids in the treatment of overweight and obesity: An update. Obes. Rev..

[9] Stohs S.J. (2013). Problems with Citrus aurantium Information in “A Review on Botanical Species and Chemical Compounds with Appetite Suppressing Properties for Body Weight Control”. Plant Foods Hum. Nutr..

[10] Brown C.M., McGrath J.C., Midgley J.M., Muir A.G.B., O’Brien J.W., Thonoor C.M., Williams C.M., Wilson V.G. (1988). Activities of octopamine and synephrine stereoisomers on α-adrenoceptors. Br. J. Pharmacol..

[11] Rossato L.G., Costa V.M., De Pinho P.G., Carvalho F., De Lourdes Bastos M., Remião F. (2011). Structural isomerization of synephrine influences its uptake and ensuing glutathione depletion in rat-isolated cardiomyocytes. Arch. Toxicol..

[12] Dusitkasem S., Herndon B.H., Somjit M., Stahl D.L., Bitticker E., Coffman J.C. (2017). Comparison of phenylephrine and ephedrine in treatment of spinal-induced hypotension in high-risk pregnancies: A narrative review. Front. Med..

[13] Stohs S.J., Shara M., Ray S.D. (2020). p-Synephrine, ephedrine, p-octopamine and m-synephrine: Comparative mechanistic, physiological and pharmacological properties. Phyther. Res..

[14] Stohs S.J., Preuss H.G. (2012). Stereochemical and pharmacological differences between naturally occurring p-synephrine and synthetic p-synephrine. J. Funct. Foods.

[15] Koh A.H.W., Chess-Williams R., Lohning A.E. (2021). HPLC-UV-QDa analysis of Citrus aurantium-labelled pre-workout supplements suggest only a minority contain the plant extract. J. Pharm. Biomed. Anal..

[16] Stohs S.J., Preuss H.G., Shara M. (2011). A review of the receptor-binding properties of p-synephrine as related to its pharmacological effects. Oxid. Med. Cell. Longev..

[17] Bloomer R.J., Canale R.E., Blankenship M.M., Hammond K.G., Fisher-Wellman K.H., Schilling B.K. (2009). Effect of the dietary supplement Meltdown on catecholamine secretion, markers of lipolysis, and metabolic rate in men and women: A randomized, placebo controlled, cross-over study. Lipids Health Dis..

[18] Ratamess N.A., Bush J.A., Kang J., Kraemer W.J., Stohs S.J., Nocera V.G., Leise M.D., Diamond K.B., Campbell S.C., Miller H.B. (2016). The Effects of Supplementation with p-Synephrine Alone and in Combination with Caffeine on Metabolic, Lipolytic, and Cardiovascular Responses during Resistance Exercise. J. Am. Coll. Nutr..

[19] Arch J.R.S. (2002). β3-adrenoceptor agonists: Potential, pitfalls and progress. Eur. J. Pharmacol..

[20] Stohs S.J., Preuss H.G., Shara M. (2012). A review of the human clinical studies involving citrus aurantium (bitter orange) extract and its primary protoalkaloid p-synephrine. Int. J. Med. Sci..

[21] Takagi M., Kimura K., Nakashima K.I., Hirai T., Inoue M. (2018). Induction of beige adipocytes by naturally occurring β3-adrenoceptor agonist p-synephrine. Eur. J. Pharmacol..

[22] Zheng X., Guo L., Wang D., Deng X. (2014). p-synephrine: A novel agonist for neuromedin U2 receptor. Biol. Pharm. Bull..

[23] Maldonado M., Bracht L., de Sá-Nakanishi A., Corréa R., Comar J., Peralta R., Bracht A. (2018). Actions of p-synephrine on hepatic enzyme activities linked to carbohydrate metabolism and ATP levels in vivo and in the perfused rat liver. Cell Biochem. Funct..

[24] Guo L.X., Chen G., Yin Z.Y., Zhang Y.H., Zheng X.X. (2019). p-Synephrine exhibits anti-adipogenic activity by activating the Akt/GSK3β signaling pathway in 3T3-L1 adipocytes. J. Food Biochem..

[25] Yun S.J., Kim E.K., Tucker D.F., Kim C.D., Birnbaum M.J., Bae S.S. (2008). Isoform-specific regulation of adipocyte differentiation by Akt/protein kinase Bα. Biochem. Biophys. Res. Commun..

[26] World Anti-Doping Agency Monitoring Program. https://www.wada-ama.org/en/resources/science-medicine/monitoring-program.

[27] Gutiérrez-Hellín J., Salinero J.J., Abían-Vicen J., Areces F., Lara B., Gallo C., Puente C., Del Coso J. (2015). Acute consumption of p-synephrine does not enhance performance in sprint athletes. Appl. Physiol. Nutr. Metab..

[28] Gutiérrez-Hellín J., Baltazar-Martins G., Rodríguez I., Lara B., Ruiz-Moreno C., Aguilar-Navarro M., Del Coso J. (2020). p-Synephrine, the main protoalkaloid of Citrus aurantium, raises fat oxidation during exercise in elite cyclists. Eur. J. Sport Sci..

[29] Ratamess N.A., Bush J.A., Kang J., Kraemer W.J., Stohs S.J., Nocera V.G., Leise M.D., Diamond K.B., Faigenbaum A.D. (2015). The effects of supplementation with P-Synephrine alone and in combination with caffeine on resistance exercise performance. J. Int. Soc. Sports Nutr..

[30] Del Coso J., Hamouti N., Ortega J.F., Mora-Rodriguez R. (2010). Aerobic fitness determines whole-body fat oxidation rate during exercise in the heat. Appl. Physiol. Nutr. Metab..

[31] Gutiérrez-Hellín J., Del Coso J. (2018). Dose–Response Effects of p-Synephrine on Fat Oxidation Rate during Exercise of Increasing Intensity. Phyther. Res..

[32] Gutiérrez-Hellín J., Ruiz-Moreno C., Del Coso J. (2019). Acute p-synephrine ingestion increases whole-body fat oxidation during 1-h of cycling at Fatmax. Eur. J. Nutr..

[33] Gutiérrez-Hellín J., Del Coso J. (2018). Effects of p-Synephrine and Caffeine Ingestion on Substrate Oxidation during Exercise. Med. Sci. Sports Exerc..

[34] Ruiz-Moreno C., Gutiérrez-Hellín J., Amaro-Gahete F.J., González-García J., Giráldez-Costas V., Pérez-García V., Del Coso J. (2020). Caffeine increases whole-body fat oxidation during 1 h of cycling at Fatmax. Eur. J. Nutr..

[35] Mata F., Valenzuela P.L., Gimenez J., Tur C., Ferreria D., Domínguez R., Sanchez-Oliver A.J., Sanz J.M.M. (2019). Carbohydrate availability and physical performance: Physiological overview and practical recommendations. Nutrients.

[36] Haller C.A., Duan M., Jacob P., Benowitz N. (2008). Human pharmacology of a performance-enhancing dietary supplement under resting and exercise conditions. Br. J. Clin. Pharmacol..

[37] Deshmukh N.S., Stohs S.J., Magar C.C., Kale A., Sowmya B. (2017). Bitter orange (*Citrus aurantium* L.) extract subchronic 90-day safety study in rats. Toxicol. Rep..

[38] Kaats G.R., Miller H., Preuss H.G., Stohs S.J. (2013). A 60day double-blind, placebo-controlled safety study involving Citrus aurantium (bitter orange) extract. Food Chem. Toxicol..

[39] Shara M., Stohs S.J., Smadi M.M. (2018). Safety evaluation of *p*-synephrine following 15 days of oral administration to healthy subjects: A clinical study. Phyther. Res..

[40] Bush J.A., Ratamess N.A., Stohs S.J., Ellis N.L., Vought I.T., O’Grady E.A., Kuper J.D., Kang J., Faigenbaum A.D. (2018). Acute hematological and mood perception effects of bitter orange extract (p-synephrine) consumed alone and in combination with caffeine: A placebo-controlled, double-blind study. Phyther. Res..

[41] Ratamess N.A., Bush J.A., Stohs S.J., Ellis N.L., Vought I.T., O’Grady E.A., Kuper J.D., Hasan S.B., Kang J., Faigenbaum A.D. (2018). Acute cardiovascular effects of bitter orange extract (p-synephrine) consumed alone and in combination with caffeine in human subjects: A placebo-controlled, double-blind study. Phyther. Res..

